# Encouraging planting in urban front gardens: a focus group study

**DOI:** 10.1177/17579139231163738

**Published:** 2023-03-31

**Authors:** RH Frost, N Murtagh

**Affiliations:** Research Department of Primary Care and Population Health, UCL, Royal Free Campus, Rowland Hill Street, London NW3 2PF, UK; The Bartlett School of Sustainable Construction, UCL, London, UK

**Keywords:** gardens, qualitative research, health behaviour, environment and public health

## Abstract

**Aims:**

Encouraging planting in front gardens offers mental and physical health benefits, as well as positive local environmental impacts such as reducing flood risk and improving air quality. However, urban front garden greenery has reduced in recent years. We aimed to explore adults’ views regarding planting greenery in front gardens, barriers and facilitators, and their understanding of health and environmental impacts, to identify appropriate intervention mechanisms for behaviour change.

**Methods:**

We carried out five online focus groups with 20 participants aged 20–64 in England, purposively sampled for variation according to age, gender, home ownership, income, ethnicity and residing in an urban or suburban area. We audio recorded each focus group, transcribed it verbatim and analysed transcripts using thematic analysis.

**Results:**

Front gardening was a relaxing activity that provided benefits including increased wellbeing, fresh air and vitamin D. Planting in front gardens depended heavily on available time and space, garden orientation, local security and the weather. Front gardens could be a place for social interaction. Participants tended to prioritise neatness and tidiness over greenery. Lack of knowledge and low self-efficacy were key barriers. There was little awareness of the environmental benefits of front garden greenery; however, reducing flood risk and encouraging biodiversity were viewed positively.

**Conclusion:**

Initiatives to encourage front garden planting should focus on plants that require little knowledge to acquire and care for, are suitable to the local environmental conditions and with a visual impact of neatness and bright colour. Campaigns should draw attention to local flood risk reduction and increasing biodiversity, in addition to personal health benefits.

## Introduction

Green spaces are an important asset for supporting physical and mental health, particularly in urban environments. They offer opportunities for physical activity and socialising and provide a healthier environment^1^ by improving air quality.^2^ This in turn reduces risk factors for poorer health such as higher body mass index (BMI) and inflammation levels.^3^ Green spaces are also positively associated with long-term mental health benefits,^1,4^ by triggering restorative undirected attention.^5^

Due to these benefits, within the United Kingdom, there has been a recent policy focus on creating and preserving green space. A 2020 Public Health England^6^ review recommended that green and blue spaces be considered critical health assets, with local strategies to develop and maintain these spaces. In conjunction with other interventions, green spaces also contribute to the national strategies for physical activity,^7^ by creating more attractive areas to exercise, and for clean air, by reducing pollution from local roads.^8^ NHS England put forward its own set of health-based recommendations in 2019 from its healthy new towns programme, including a focus on encouraging green spaces including private gardens and street greenery to be included in development.^9^ This is particularly vital for deprived areas, which generally have less access to green space.^6^

Vegetated private garden space is a key part of local green infrastructure in the United Kingdom – 88% of British homes have access to private garden space.^10^ The People and Nature representative survey of 24,994 adults across England found that 79% spent time in private gardens at least once a week, a much higher figure than 49% who had visited a community green space within the last month.^11^ Increased time spent gardening is associated with greater vegetation in front gardens,^12^ and gardening activity is linked to improved health outcomes,^13^ reduced risk of vitamin D deficiency,^14^ increased fruit and vegetable intake and lower BMI.^15^

However, there has been little focus in previous studies on front gardens, with many studies focusing on food production with assumptions of adequate space, security and privacy. Urban front gardens, the space in a residential dwelling between the front of the house and the street, are a particularly under-utilised and often small space. Although considered private, front garden spaces are seen by and may impact upon passers-by as well as those dwelling in the home. This contrasts to back gardens, which as they are placed at the rear of the house, are usually hidden from view, except for immediate neighbours. Higher-quality street greenery is associated with better perceived health, better mental health, and reduced acute health-related complaints, while higher quality and quantity of street greenery are associated with reduced stress and increased social cohesion.^16^

Front garden greenery offers additional indirect health benefits through environmental services, including reducing local flood risk,^17^ cooling the home in hot weather and reducing air pollution from the street.^18^ There are further benefits from vegetation and soil to carbon sequestration and to supporting biodiversity. It is therefore important to encourage the activity of gardening specifically in front gardens, to increase the level and quality of street greenery as well as ecosystem service co-benefits. Simple interventions such as introducing a small number of potted plants to front gardens in deprived areas show reduced stress, improvements in salivary cortisol parameters and increased sense of pride and care in the street.^19^ While funding arrangements are needed to preserve local green space,^6^ private garden spaces do not require local authority maintenance in the same way as other community green spaces. This further aligns with an asset-based public health approach of mobilising community assets and maximising people’s ability utilise these, in order to support individual and community health and wellbeing.^20^

However, private garden spaces (particularly front gardens) are more vulnerable to loss after development, from the changes and preferences of individual owners. In recent decades front garden greenery has reduced, with more hard standing and car parking space being introduced. A London study estimated that impermeable surfaces comprised almost two-thirds of front gardens, with a 40% loss of lawns in the previous 9 years.^21^ This reflects a global change, with countries such as Germany and India also reporting loss of urban garden space.^22,23^ Given the consequent health and environmental impact, it is vital to understand what affects individuals’ choices about greenery in their front garden, and how to encourage gardening and planting of greenery. It is particularly timely to focus on private garden space, as during the COVID-19 lockdowns citizens in many countries were confined to their own homes, with limited outdoor time except within their own private garden. This highlighted the importance of private green space for fresh air, activity and limited social interaction with neighbours.

Urban gardening interventions have typically focussed on green social prescribing in community gardens,^24^ community gardens and health outcomes,^25^ or fruit and vegetable growth for improved nutrition.^26,27^ Few interventions have been developed to increase home gardening and planting behaviour. This study was informed by an established model of behaviour change (COM-B), widely used in health psychology.^25^ COM-B (Capability, Opportunity, Motivation, Behaviour) sets out the three types of factor necessary for behaviour to occur: the physical and psychological capabilities to undertake a behaviour, the social and physical opportunities and the automatic and conscious motivations.^28^ COM-B forms part of a theoretical framework, the Behaviour Change Wheel, which maps intervention functions on to these components to overcome specific barriers and enable behaviour change.^28^ The framework offers a rigorous and systematic way to understand the range of factors affecting front gardening behaviour and subsequently to identify appropriate intervention types to target the behavioural determinants.

Surveys from a range of countries indicate that motivations for gardening are varied, such as aesthetics/sensory reasons, spending time outdoors, shading the house, observing nature and relaxation, pleasure or hobby, as a source of food, health, and seeing plants grow.^12,29,30^ However, these studies surveyed wholly or predominantly gardeners, with samples skewed on gender and potentially on age and income. With the exception of one recent UK research programme,^12^ these studies do not differentiate between front and back gardens. As quantitative surveys, they were unable to capture nuance and depth of experience or context. A gap remains on understanding the motivations of the broader population relating to why they choose, or do not choose, to garden in their front garden space.

We therefore carried out a qualitative research study to explore adults’ views about planting greenery in front gardens, barriers and facilitators, and perceived associations between front gardens, health and wellbeing.

## Methods

Focus groups use group interaction to understand what and how participants think, including shared understanding and norms.^31^ We carried out five online focus groups, each with four participants, in February and March 2021 in the United Kingdom using commonly available videoconferencing software. At the time, the United Kingdom was under a third national lockdown due to the COVID-19 pandemic, with the public advised to stay at home and closure of non-essential businesses. We recruited adults aged 20–65 years, resident in England in urban or suburban areas, with a ground-floor front garden space between their door and the street at least the size of three large waste bins. Participants were purposively sampled for gardening/not gardening in their front garden, as well as potentially relevant demographic characteristics: renting/home ownership, ethnicity, income, gender and age. Participants were recruited and consented through a market research company and were paid £40 each for participating in a focus group, in order to encourage non-gardeners and underserved groups to participate. The study received ethical approval from University College London Bartlett School of Construction and Project Management Ethics Committee (ref 2020-StF-NM-002).

We developed the topic guide based on the research question, previous literature and the COM-B model.^28^ Topics covered included the use and function of front gardens; and motivations, opportunities and capabilities required for planting greenery at the front and barriers to this.

We planned focus groups for gardeners (n = 1), non-gardeners (n = 1) and mixed (n = 3), with slightly adapted topic guides. As we were particularly interested in physical health and environmental factors and these were not usually spontaneously raised, we also introduced information and questions on this in a neutral way such as ‘Planting in front gardens can reduce your local flood risk. Is that something you’ve ever thought about when deciding to plant greenery in your front garden?’. Focus groups were led by RF (a health sciences researcher) and co-facilitated by NM (an environmental psychologist), both with expertise in qualitative research. They lasted 64–84 min.

Focus groups were audio recorded and transcribed verbatim. We carried out codebook thematic analysis.^32^ All transcripts were read, and both authors independently generated an initial coding framework, which was amalgamated through discussion into a single framework and applied in a second round of more detailed coding. Although our coding framework was organised around the COM-B model,^28^ to understand the behaviour of planting greenery in front gardens, we developed themes inductively, rereading data under each code and re-organising them into health-related themes, refined iteratively through writing.

## Results

Our 20 participants were varied with regards to gender, geographic region, home ownership, location and characteristics of front garden spaces, with some variation in ethnicity and age and limited variation in income (see Table 1 and Box 1). Dichotomising participants into gardeners and non-gardeners was more difficult than anticipated as participants described varied levels of gardening participation: the range of these experiences is reported throughout the results.

**Table 1:** Demographics of study sample (n = 20).

Focus group type	Gardening status	Demographics
Gardeners	4 gardeners	2 male, 2 female 2 × 50–64 years, 2 × 35–49 years 2 suburban, 2 urban 2 owners, 2 renters 4 White British Income 2 × <£30k, 1 × £31–50k, 1 × £50k+
Non-gardeners	4 non-gardeners	2 male, 2 female 2 × 50–64 years, 2 × 35–49 years 2 urban, 2 suburban 2 owners, 2 renters 4 White British Income 2 × <£30k, 2 × £31–50k
Mixed 1	2 gardeners 2 non-gardeners	2 male, 2 female 2 × 35–49 years, 2 × 50–64 years 2 suburban, 2 urban 4 owners 4 White British Income 1 × <£30k, 3 × £31–50k
Mixed 2	2 gardeners 2 non-gardeners	3 male, 1 female 3 × 35-49yrs, 1 × 20-34yrs 3 suburban, 1 urban 3 owners, 1 renter 2 Asian/Asian British, 2 White British Income 1 × <£10k, 2 × £10k-31k, 1 × £31-50k
Mixed 3	2 gardeners 2 non-gardeners	1 male, 3 female 1 × 20−34 years, 2 × 35–49 years, 1 × 50–64 years 2 suburban, 2 urban 3 owners, 1 renter 1 Asian/Asian British, 1 Black African/Black British, 1 White British Income 3 × £10–31k, 1 × £31–50k

**Box 1:** Characteristics of front garden spaces.

We sampled participants with a diverse range of front garden spaces. Sizes varied from a “very small little patch” (Mixed 2) to “quite a large front garden really. . .we’ve got a corner plot on a detached house at the end of a cul de sac.” (Mixed 1). The content of front gardens also varied highly. A few had fronts with no greenery: *just tarmac. It’s literally a car park (Mixed 3)* Many described a combination of paving and greenery, with space for parking one or two cars, but with additional greenery such as bushes or trees. *it’s mostly lawn with a driveway and I’ve got a border under the front window. Massive Leylandii hedge. Another big kind of what looks like a rocket Leylandii bush, and a birch tree (Mixed 1)* Some participants had lawns, of varying sizes. A few reported hanging baskets, herbs or garden ornaments. Pots of plants were commonly discussed in front gardens: *what we have planted in the front are the two plants that were dug out but now in pots, and an olive tree in a pot. (Mixed 3)*

We identified four main themes in relation to the impact of front gardens on health: (1) effort and reward, (2) connecting with outdoor spaces, (3) the social nature of front garden spaces, and (4) gardening knowledge and self-efficacy.

## Effort and Reward

The most salient benefits of front garden greenery related to mental wellbeing and occupational activity, with gardens described as ‘therapeutic’ and ‘a sanctuary’. Part of this related to being outdoors in pleasant surroundings, but more often people related this to activity, with the idea of ‘pottering’ raised in most groups.

*I love like mowing the lawn and doing things like that. . .some days I’ll just potter and prune things back. (Gardeners)*

This was particularly the case for those who expressed greater enthusiasm for gardening, for both front and back gardens. Gardening was viewed as an absorbing distraction from stressors that led people to focus on the immediate present and ‘forget about the world’ (Mixed 1). This led to increased wellbeing.

*You could genuinely switch off because you [are] just digging mud. (Mixed 1)*

Others enjoyed gardens as a source of projects and creativity. The act of planning and seeing results generated pleasure, satisfaction and ongoing motivation. However, the reward aspect was less salient for front gardens than back. Those who preferred spending more time in the back garden took a utilitarian approach, calculating a low benefit to themselves of a pleasant front garden versus the effort, time and money required:

*Why would you spend loads of money making my frontage look really pukka [excellent] when I’m not the one sat looking at it (Mixed 1)*

Front gardens were also considered more vulnerable to security risks than back garden spaces, leading to less time and investment. Theft was a particular concern:

*Last summer we had people stealing hanging baskets, like [participant] said earlier. . .you put a lot of effort into growing them and making them look nice and then people stealing them for their own pleasure or whatever, it’s not really nice (Mixed 2)*

No participants who rented discussed constraints in planting greenery from landlords. However, planting was considered a financial risk, particularly given the large array of possible plants and the need for knowledge regarding what would flourish best in their particular front garden.

*I could spend 200 quid [pounds sterling], and then a month later be looking at a big brown mess. (Non-gardeners)*

In addition, for those less interested in gardening or with little free time, gardening represented a non-essential investment of time they did not have, mainly due to work and childcare. The ideal compromise for those with little time was greenery requiring little effort to maintain. Here it was advantageous if front garden plants were left by previous owners, as most were retained out of ease:

*I’m just not particularly green fingered, and it was some quite nice shrubs that I inherited. So I’ve just kind of left them to do whatever they do (Non-gardeners)*

### Gardens as outdoor spaces

The physical health benefits of gardening were chiefly related to being outdoors in the ‘fresh air’ and getting vitamin D and sunshine, with exercise benefits only acknowledged in relation to heavy lifting. Appreciation of time spent outdoors had increased during lockdowns, and for some, the front garden became another space to use, almost an outdoor room. The sensory impact of being outdoors and of plants were consistently raised. The visual impact of the front garden was a particularly strong element, with ‘colour’ from flowering plants and ornaments valued highly as a key element of front gardens, both for one’s own and others’ benefit:

*If you’ve got it looking nice and tidy and full of different colours and plants and stuff, it’s inviting (Mixed 2)*

Those without front garden greenery regretted having a less visually appealing garden, but rarely discussed other sensory aspects. Scents, such as freshly cut grass or lavender, were mentioned mainly by gardeners. However, the outdoors was not always experienced as pleasant – there was strong consensus in one focus group that litter (deliberately deposited or blown in by wind) was an issue, while insects, cat mess and hayfever were mentioned by a small number of participants as particular front garden issues. Poor weather was also a key barrier both to gardening and front garden greenery.

Front garden use depended heavily on orientation and position. Sunshine available in the front garden influenced time spent there and the plants that could be grown, while size influenced both what could be planted and whether a larger back garden space was more often used. Those near a main road felt it would be less pleasant to sit or garden at the front. Space for parking was a common issue, where need for off-street parking took precedence over greenery:

*It was never an option to turf it or grass it or garden it purely because the girls were getting older and driving and we needed the space for cars (Mixed 3)*

Despite appreciating outdoor time, planting greenery in front gardens for environmental reasons was not spontaneously raised in focus groups. Both gardeners and non-gardeners treated the idea with surprise and thoughtfulness, demonstrating a disconnect between front gardens and local environmental impacts:

*I’ve never really considered [reducing flood risk], but it actually makes sense (Non-gardeners)*

Enthusiasm was however expressed for learning more and raising awareness of ways in which front garden planting could have a tangible local impact, with particular emphasis on reducing flood risk locally and promoting biodiversity.

*if someone says if you plant this, it would help the bees then, or encourage the bees, I would say ‘yeah ok’ because I like the idea (Non-gardeners)*

### The social nature of front garden spaces

Front gardens had clear social benefits. There was a strong consensus within and across focus groups that spending time on activities in the front garden was an open invitation for neighbours and passers-by to chat, which many welcomed:

*In the front garden, you chat to people (Gardeners)*

Plants could be an important connection with friends and family, particularly for those more interested in gardening. A few mentioned that plants given to them by someone who had since passed away acted as visible reminders of the person. In all focus groups, some of the participants reported others starting conversations about plants or exchanging plants between family, friends and neighbours.

Lockdowns during the COVID-19 pandemic had increased the salience of front gardens as social spaces that developed a stronger sense of local community, offering incidental socialising opportunities for those spending the majority of their day at home, or as outdoor visiting spaces. Victory in Europe (VE) Day street parties (where people celebrated in their front gardens at a social distance) were spontaneously recalled as a key example of this. Increased socialising did depend highly on relationships with individual neighbours. Furthermore, there was a perceived need to be active in the front garden space, with the idea of sitting and relaxing out the front holding a stigma for some:

*I think sitting out the front people would say either this person’s got too much time or he’s looking at the neighbourhood gossip (Mixed 3)*

Relaxation was seen as something to mainly do in more private spaces, such as the back garden, partly as relief from socialising. Due to the high visibility of front garden spaces, participants considered how they represented their own social identity in the neighbourhood, and made positive and negative judgements about neighbours based on their front gardens:

*us and my two neighbours fortunately do tend to put a lot of time and effort into their garden, but then others are. . . There’s a washing machine, a sofa and a mattress sitting in the front. . .They’re obviously going to have no pride in anything. (Mixed 2)*

Being ‘neat and tidy’ was therefore prioritised as the ideal front garden, which could sometimes contrast with the idea of having lots of plants and greenery.

*A nice, neat, neat lawn and a nice driveway, I think it looks good. But it’s not necessarily about loads of plants. I think it’s just tidiness (Mixed 1)*

Few participants discussed wilding approaches to gardening, but where they did, they themselves considered it untidy or believed their neighbours would. Simple garden features with easy maintenance were valued, such as a lawn or pots. People also felt there was more pressure on front gardens to fit in with the rest of the street, suggesting that street-level initiatives could be useful.

### Knowledge and self-efficacy for planting

Those who gardened had typically accumulated knowledge over many years and were strongly interested in gardening. They often used more specific language, discussing ‘perennials’, ‘bedding plants’ and specific species, terminology that was off-putting for non-gardeners:

*if you say bedding plant, I don’t really understand what that means. . .I just want plants that stay green all year round and don’t drop their leaves (Non-gardeners)*

Jargon was particularly intimidating when visiting a garden centre, with some participants reporting embarrassment about their lack of knowledge and finding signage difficult to navigate. In contrast, more confident gardeners spoke about their local garden centres as a very helpful source of information. Gardening knowledge was seen as something primarily gained from experience and trial and error. Less confident gardeners typically relied on knowledge and advice, or gardening itself, from more expert partners or parents. There was a strong intergenerational quality to gardening – most participants (whether non-gardeners or gardeners) had learnt about gardening through family members, often parents or grandparents:

*I’d never known and never needed Alan Titchmarsh or Charlie Dimmock [UK TV gardening show presenters] to show me to tap and pull the roots out to encourage it. I’ve learned that from my grandparents. (Gardeners)*

People were therefore the most important gardening resource, with ongoing exchanges of ideas, plant cuttings and advice. On this basis, most focus groups favoured school-based interventions to engage young people in lifelong gardening and to teach basic principles. TV shows were potentially useful but criticised for concentrating on large-scale complex landscaping projects rather than simple basics achievable in limited spaces. Books were used by some, while websites were seen as an easy way of getting answers to specific questions and ideas for ways to change gardens.

## Discussion

Front gardens were valued as spaces that improved wellbeing through relaxing activity, visual benefits, socialising and through being outdoors in the fresh air. However, participants were only willing to invest time, money and effort on the space if, first, they spent a lot of time in it, which depended heavily on their time commitments, garden orientation, weather and local environmental factors such as litter; and second, if they felt sufficiently confident that they would see good results. Front gardens presented a social image to others, but were rarely connected with local or global environmental benefits. Basic knowledge and self-efficacy for planting were key factors affecting whether people planted greenery. Participants mainly learnt gardening through parents and grandparents, reinforced by trial and error, others’ advice and the Internet.

However, there are few initiatives focusing on front gardens as a key area for change and how we can encourage individual behaviour change on this topic, despite their value as an individual and community health asset. Table 2 maps barriers and facilitators discussed in each subtheme to the capability, opportunity and motivation dimensions of the COM-B framework. We have mapped these to the intervention functions from Michie *et al.*^28^ and to interventions reflecting these functions that were suggested by our participants. The range of barriers identified in Table 2 provide a starting point for local intervention – organisations can identify which are most relevant barriers for a particular population or area, what can be changed and at what level. When planning interventions, a multi-pronged approach addressing barriers across capability, opportunity and motivation are necessary to succeed in initiating behaviour change, particularly when interventions are co-designed. Implementing interventions at different levels (e.g. mass communication, local policy, and local initiatives) will facilitate these processes.

**Table 2:** COM-B breakdown of factors affecting front gardening, associated intervention functions and how this could be implemented.

Factors identified from qualitative analysis	Intervention functions from Michie et al.^28^	Examples of how this could be applied based on focus group data
Capability Psychological • Knowledge (from experience, learning from previous generations) • Self-efficacy and confidence Physical • Physical barriers were not mentioned by participants.	Training Enablement	Providing information on gardening in front gardens and simple basics through websites, TV and books. Maximise information available in garden centres, with clear directions to match garden conditions to plants available. Provide different levels of information targeted to different audiences (e.g. novice gardeners) Imparting skills through events and programmes such as local fairs or gardening clubs Encouraging learning from a young age between parents or grandparents and children, or school-based interventions Encouraging people to start with small changes and build up
Opportunity Physical • Time available • Resources (financial risk) • Inherited plants • Weather/climate • Requirement for other use of space (mainly parking) • Characteristics of garden (orientation, position etc) • Location of front garden (e.g. near main road) Social • Social norms of family • Social norms of street • Front gardens as reflection of social identity	Restriction Environmental restructuring Enablement	Change planning regulations to ensure new homes are built with greenery in the front garden Encourage large landscaping companies to consider environmental impact of changes Providing plants Provide advice on low-cost gardening and planting in different garden conditions and locations Provide advice on or supply plants that require little effort to maintain Encourage local in person and social media networks on gardening tips and exchanges of plants Encouraging spending time gardening between parents or grandparents and children Promoting pride in local neighbourhood
Motivation Reflective • Improving biodiversity • Reducing flood risk • Look nice and tidy for others • Security and litter • Frequency of use Automatic • Sensory benefits • Mental wellbeing • Fresh air and vitamin D • Socialising • Features/plants with emotional connections	Education Persuasion Incentivisation Coercion Persuasion Incentivisation Coercion Environmental restructuring Modelling Enablement	Promote the health, wellbeing, environmental and social benefits to both gardening activity and the end results of planting in front gardens Link to wildlife (e.g. supporting bees) and clear benefits to the local environment Provide an emotional reason to plant something (e.g. encouraging plants as gifts, planting as a memorial) Council grants, vouchers or other incentives Local competitions for front gardens or streets Provide examples of similar front gardens that have been transformed to be more green and visually appealing

Gardening in general has received previous attention and study – positive personal effects on physical and mental health have been extensively demonstrated.^13–15^ However, our study showed that mental health benefits and personal enjoyment are more strongly prioritised than gardening as physical activity, aligning with an earlier large-scale quantitative study,^12^ and so these should be communicated more widely.

Our research showed that the broader value of front garden spaces needs to be promoted at both national and local levels. Given the opportunity barriers to change identified in our study, local initiatives are needed to encourage green front gardens when planning new housing or to encourage change through incentives or neighbourhood projects. Schemes such as Britain in Bloom, an annual national competition which encourages planting and tidying in local areas,^33^ shows positive community, health, economic and environmental impacts.^34^ At present, this focuses mainly on community spaces rather than private gardens, but this could provide a useful template for further national or local strategies, for example, greenest street competitions. The current campaign of the Royal Horticultural Society (the United Kingdom’s major gardening charity),^35^ Greening Great Britain, includes a focus on front gardens from a national perspective; however, there is a need to ensure this is disseminated more widely and translated into local campaigns, projects and strategies. These will benefit from further qualitative research or co-design approaches with residents to ensure they respond to the local context, as well as applying the insights on motivations and barriers outlined above.

These campaigns could also raise awareness of wider social benefits, as contributing to a nicer street or creating a pleasant space for active travel were rarely discussed, with participants placing greater focus on how their front garden reflected themselves, rather than the local community. Likewise, a national UK survey found neighbours and community were mentioned as a reason for gardening by <5% respondents.^12^ Social benefits were seen as important in this study but more about creating connections between neighbours and generations than providing a green community environment or local or global environmental benefits, so campaigns could encourage providing plants as gifts. Neatness was prioritised in a UK context in our study, with participants generally preferring a low-effort garden unless they were interested in improving their garden as an ongoing project. Previous work has found that greenery quality (variation, maintenance, orderly arrangement, absence of litter, and general impression) better predicted health, stress and social cohesion than quantity,^16^ suggesting even small changes to promote greenery that maintain a neat and tidy front may be beneficial. Policymakers should be aware that encouraging changes in front gardens may have long-lasting effects, as people often kept plants from the previous homeowner, and may trigger further changes in the community as people feel a social pressure to fit in with the rest of the street.

Perception of self-efficacy for gardening needs to be addressed – it was clear from some non-gardeners in our study that ‘mistakes’ and feeling unable to understand gardening jargon could significantly affect confidence. Initiatives to encourage planting in front gardens should focus on simple, cost-effective methods to increase planting that have a visual impact of neatness and bright colour. Clear instructions or recommended plants that are suitable for the terrain may help to overcome initial self-efficacy and environmental barriers and build positive reinforcement. Although there is existing information and resources – for example, the Royal Horticultural Society website allows someone to find plants based on garden conditions and has space for planning your own garden,^35^ wider awareness and promotion of these kind of resources is needed. Social media may also play a role in this.

While many of the suggested behaviour change interventions from our COM-B analysis rely on education and advice, important opportunity barriers were also detected such as garden orientation, position and factors such as resources, security and litter. These may require more active local strategies, such as providing low cost or free access to suitable plants, implementing planning regulations regarding green space in front gardens and ensuring streets are well-maintained. These interventions and initiatives to encourage planting in front gardens need to be evaluated to build up an evidence base, particularly with regards to long-term effects. This approach is likely to apply across countries and contexts, with specific consideration given to likely variations in cultural norms, climate for growing plants, available housing stock and planning regulations.

Limitations of this study include a lack of patient and public involvement, although representatives were included in other aspects of this project. This article provides a starting point for exploring this topic in the UK context, and future studies are needed to explore subgroups in more depth (e.g. other countries within the United Kingdom, certain types of residence, those on low incomes). This was carried out in England, and while the findings align well with international survey studies,^29,30^ more qualitative work is needed in different cultures and climatic zones. This was also carried out within the context of a national pandemic where people had been instructed to stay at home. Further work is needed to explore whether there have been shifts in front gardening behaviour or motivations ascribable to or since pandemic lockdowns. As this is a qualitative study, the study was not intended to quantify or determine the relative importance of each of the barriers and facilitators to the wider UK public, which remains an area for further research using different methodologies.

## Conclusion

In conclusion, initiatives to encourage planting in front gardens are likely to be most successful if they focus on plants that are easy to access, simple to care for, do not take up too much space, are suitable to the environment and have a visual impact of neatness and bright colour. Campaigns to encourage planting greenery in front gardens should highlight the specific benefits of front gardening, particularly to reducing local flood risk and increasing biodiversity, in addition to local community and health benefits.
